# The Milk Supply of Atlanta; the Dangers Existing in Impure Milk, and Remedy

**Published:** 1907-06

**Authors:** L. B. Clarke

**Affiliations:** Lecturer on Diseases of Children, Atlanta School of Medicine, Atlanta, Ga.


### THE MILK SUPPLY OF ATLANTA; THE DANGERS EXISTING IN IMPURE MILK, AND THE REMEDY.*

By L. B. Clarke, M. D.

Lecturer on Diseases of Children, Atlanta School of Medicine, Atlanta, Ga.

This paper is intended not alone as a criticism and arraignment of the fearful conditions that have for some time past, and until very recently, existed in our local milk supply, but additionally, to point out how contamination occurs, and argue for more restrictions that will insure a cleaner, safer, purer milk supply, demonstrating how this is accomplished in other cities in the production and handling.

I have conceived this duty to fall peculiarly to the lot of a teacher of diseases of children, since, in our medical colleges to-day, one of the most important features of this branch is a thorough

*Read before the Fulton County Medical Society, April 25, 1907

course in the feeding of infants artificially, and a study of milk purity and contamination.

A paper dealing with these questions as they affect children, has been in view and in mind for more than a year, it being the writer's intention to bring this matter before this society for the purpose of taking hold of the question. I have been forestalled, however, by the city board of health, which has been awakened to the true state of affairs, by the city bacteriologist, and an effective and much needed crusade has been begun.

The proper food for nursing infants is mother's milk. In the event that this is impossible, for one or more of the various reasons that may render it so, the substitute used in the greater part of the world is cow's milk, which is also the chief article of diet for nearly all young children and for many adults.

How essential, therefore, that the food upon which a large number of the people of this city depend for subsistence, should be pure and clean, neither chemically adulterated nor contain preservatives to carry it beyond its ordinary period of freshness, thus retarding its souring, nor bacterially infested with any or all of the disease producing organisms that exist.

How Atlanta's milk supply has fallen woefully short of this standard, we will attempt to show.

With a flourish of trumpets, a beating of cymbals, a picture on their delivery wagons supposed to be an infant with an overplus of adipose tissue, and much to say in the way of lulling the people into a sense of false security with the word—"pasteurization," a certain milk selling company made its advent in this community about 2 years ago. For the past year the writer has encountered complaint after complaint from his patients as to the character and cleanliness of the milk this company was selling, and in each instance discontinued buying of them.

One mother whose child was under my professional care, asked if they were selling real milk; that she could not conceive that the stuff was real milk: that she could not keep it at all. Another rendered the same complaint: she could not keep it on ice but a few hours before it spoiled, could it possibly have been fresh when delivered to her? Another stated that the milk would sour, but would not clabber.

I have personally seen not alone these, but other dealers driving about the city with milk "sloshing" about in tin cans. I have seen buttermilk open in tin cans, with an excuse for a cover in the shape of a piece of paper, which had once been on the can.

During my investigations the following came home to me. I had been buying milk of a splendid and excellent quality from C.— an expert dairyman from Michigan. During the absence of my family from the city for 3 months last summer, I discontinued, and when upon their return I communicated with my dairyman with a view to again obtaining his milk, I learned he had sold out to the larger company just mentioned. I, of course, refused to follow. A few weeks later, seeing one of the old C. wagons pass my house, I stopped it, and inquired of the young white lad, whose milk he was selling, answer, "said company."

How is it then, that you are using C's wagon? "Well," answered the unsophisticated lad, "we have C's old customers who won't have any but C— milk, and won't buy it from the regular wagon." Those customers knew, they were informed, and still the nefarious business went on. The question arises here:—Whose milk were they selling to the old C— customers under the —C brand?

The worst and most unpardonable feature of this business, however, was the selling of impure milk, around which the misleading cloak of pasteurization, had been thrown, and this pasteurization done on milk produced at distant points, brought here and submitted to pasteurization after being many hours old, as if that destroyed the toxines existing and generating in the milk since the milking.

I have been informed by a lady whose children I have attended, that she has seen another dairyman refill bottles that he had collected from a front porch with milk from a large can on his wagon, and leave it at the next house. How clean were those bottles, left exposed and uncovered on a front porch near the street for hours, probably from the night before?

A patient of mine walked into a dairy depot upon some matter of business one day, and it chanced to be the dairy from which he received his milk supply. He went behind the scenes and decided that he would change, preferring to have milk on his table before the flies had their bath.

Now, what are the dangers arising from feeding impure milk, especially to infants and children? They are both immediate and remote. We will first discuss the immediate.

DIARRHŒA.

The feeding of impure milk is one of the most potent causes, especially during the summer months. Investigations a few years since conducted by the Rockefeller Institute developed the information that in New York City, of nearly 600 bottle-fed infants living in the tenement districts, the death-rate in summer was 10 1-2 per cent. and of this, 80 per cent. was due to diarrhœal diseases produced by bacteria in impure milk. These figures apply to bottle-nursing infants. Only 3 per cent. of diarrhœal deaths occur in breast-fed babies. Death is rare.

"SUMMER COMPLAINT." GASTRO-ENTERIC INTOXICATION—GASTRO ENTERITIS.

There are many causes of this disease in children, yet the form produced by acute milk poisoning results in severe, sudden toxic symptoms, which are classed amoung the most dangerous forms of infection, so much so, that milk considerations are carefully gone into, in the prophylaxis of this disease. The prevention of this disease, recognizing the rules laid down by all authorities, consists among the most prominent instructions, in watching with unremitting vigilance the kind and quantity of food taken by the child and exercising the most rigid precaution in the care, methods of transportation and handling of milk. The mortality has been wonderfully reduced in all the large Eastern centers where either, from private generosity or through Health Boards, a pure Milk Supply in sealed bottles has been available. The philanthropic work in this line done by Mr. Nathan Strauss in New York is notable.

CHOLERA INFANTUM.

This much-dreaded contributor to infantile mortality is the most important of the entire series.

The disease is closely and intimately associated with impure milk, more so than the other intestinal diseases of childhood. Indeed, impure milk is the one recognized cause of cholera infantum, which is produced by poisons pre-existing in the milk or developing after being taken into the stomach. The death rate in cholera infantum is about 70 per cent. If this were the only disease in existence produced by impure milk, the restrictions asked for at the conclusion of this paper would be fully warranted.

ILEO-COLITIS—DYSENTERY.

In some of its forms is often distinctly traceable to milk infection. The catarrhal types, and especially the Follicular type, so often the outcome of a Gastro-Enteric intoxication, are commonly produced by infected milk.

The actual relation existing between impure milk and the "Shiga Alkaline" or "Flexner Acid" Bacilli Dysentericæ is at present not thoroughly known.

SOME LOCAL FIGURES ON INTESTINAL DISEASES.

Our local statistics for the year 1906, shows as follows;

| | White | Col. | Total. |
|---|---|---|---|
| Total deaths under two years, all diseases ........ | 258 | 272 | 530 |
| Of these due to all bowel diseases .............. | 74 | 59 | 133 |

Or 25 per cent of all. Of these, 74 white, 80 per cent (59), occurred in summer months, May to September, and of these June was the heaviest month, 24 deaths. Of the 59 colored, 46 occurred in summer, May to September, with July as the heaviest month, 16 deaths.

These figures apply only to children under two years.

In Atlanta statistics, from two years to old age are classed in one list.

Consider the vast increase in this mortality, if the figures for other ages were included, especially children from two to five years.

And these are to a great extent preventable diseases, because impure milk is the greatest cause of all of them.

PECIFIC ORGANISMS. CONTAGIOUS AND INFECTIOUS DISEASES

TUBERCULOSIS.

Koch's theory that bovine and human tuberculosis differ from each other and the transmission not common, has been exploded by the results of a series of exhaustive researches conducted by the U. S. Dept. of Agriculture and likewise by a commission in England. The results obtained warrant the strong presumption that infection with tuberculosis occurs much oftener by the digestive tract than through the respiratory passages, and cow's milk the most common source of infection, and while pasteurization may be the practical method by which to destroy the bacilli, the more common use of the tuberculin test is demanded as the best and most radical means of eradication.

Tuberculosis, occurring through the alimentary canal becomes easily preventable.

The leading veterinary surgeons state that tuberculosis in cattle is steadily increasing in the United States and estimate that in N. Y. fully ten per cent. of the dairy cows are tuberculous.

In Penn. 2 to 3 per cent and in the Eastern States generally from 10 to 25 per cent. In London cattle 25 per cent are found in the slaughter houses. Bacilli may not be found in the milk until the disease is well advanced, or the disease chances to be located at the udder.

The remaining diseases communicable from the cow direct are Anthrax and Foot and Mouth disease.

TYPHOID—SCARLET FEVER—DIPHTHERIA.

A few years ago, from the world at large were collected statistics regarding contagious diseases, and nearly 350 separate epidemics ascertained to have been spread through the agency of milk.

It readily, easily, greedily becomes infected. Of these outbreaks mentioned:

"One hundred and ninety-five epidemics (not cases) were typhoid fever".

"Ninety-one epidemics were scarlet fever."

"And the remainder were diphtheria."

Typhoid fever knows no greater agency of spread. Witness the experience in this city last year from the milk supply.

"Rigid search into the methods of disseminating the diseases enumerated in the outbreaks just mentioned, developed one or other of these causes.

"1. The disease existed at the dairy.

"2. Milk diluted with infected well water.

"3. Cows wading in filthy water.

"4. Employees nursing contagious disease at home and continuing to work at the dairy.

"5. Employees themselves with disease in mild form and continuing at work.

"6. Milk utensils washed with infected cloths.

"7. Milk kept in closets in sick rooms.

"8. Employees of dairy visiting at infected homes.

"9. Refilled cans or bottles from houses of patients.

REMOTE RESULTS OF IMPURE MILK.

Scurvy and Rickets.—If milk be so impure that instead of feeding the infant what its proper development of bone, muscle, blood and nerve structure demands—fresh food.—it is necessary to sterilize that milk, because of its taint, then be it known that of the causes of scurvy sterilized milk ranks second in prominence.

In a series of investigations into over 400 cases of scurvy conducted by the American Pediatric Society, 107 of these cases, exactly 25 per cent. were found due to sterilized milk.

Rickets, while produced from dietetic errors, chiefly excess of carbo-hydrates and great deficiency in fat and proteids, was nevertheless associated in the scurvy investigation to the extent of 45 per cent. of all cases.

I shall now pay my respects to sterilized and pasteurized milk and bring out the objections to adopting these plans for the purpose of overcoming the impurities existing in raw milk.

Sterilized milk is not bacterially sterile, not rendered so by any of the methods commonly used. It would require heating for a much greater length of time, which must not be done. It is bad enough as it is. Sterilizing by the present processes destroys pathogenic organisms, nearly all non-pathogenic, and retards certain fermentative changes, but is this all that occurs? By no means. If heating continues for one and one-half hours at 212 degrees. spores are not killed and the safety of the milk is imperiled by the fact that the milk may become dangerous at certain temperatures through development of spore-bearing bacteria. Again, all bacteria do not act upon milk-sugar, but some upon proteids, and the changes may not be apparent, because it may not sour.

What else is accomplished in sterilizing milk?

1. Constipation.

2. Casein made less coagulable by the rennet. Pepsin and trypsin action delayed.

3. Many chemical changes, viz.:

Changes in the casein.

Change in the phosphorus into inorganic phosphate.

Change in citric acid; lime salts made insoluble.

4. Fat changes.

5. Destruction of natural ferments existing in fresh milk, of value in digestion and nutrition. Scurvy proves the change in nutritive value. All cases due to sterilized milk are cured by stopping the sterilizing process.

Is this desirable food for an infant?

PASTEURIZED MILK.

Heating milk to only 150 degrees for 20 to 30 minutes, (earlier 167 degrees was used), does away with many of the objections to sterilized milk. Bacilli of Tuber. Dipth. and Typhoid are destroyed and likewise nearly all other bacteria. Thus the changes that occur in sterilizing at 212 do not take place at 150 degrees, but spores are not affected, toxines are not destroyed. The living organism may be destroyed, but the toxines produced by them remain intact and may render milk poisonous, which is thought protected by late pasteurization. If done, it must be at once Milk produced 30 miles from Atlanta and pasteurized here is not properly protected. Again, sterilization is a very simple process, pasteurizing very complicated and intricate, requiring care, special apparatus and an intelligent operator, and can not be readily done at dairies.

The lesson is simple, what we need for our babies is pure, clean, fresh, raw milk, requiring neither process.

In this city the nearness of the farms to the city (average 10 to 12 miles) will easily permit the ready delivery of perfectly fresh milk requiring no heat to protect it.

Are not these statistics and figures alarming? And is it not time that these matters were taken rigidly in hand, when we consider for a moment what we subject these little ones to by our apathy, lethargy, even neglect?

The chief trouble lies in the fact that the questions of surrounding the care of the cows, the milking, and the subsequent care of the milk, with the proper safeguards, have been considered beneath the dignity of the physicians' learning, and have been relegated to the dairyman, who has either wilfully neglected, or has been woefully ignorant of the causes and remedies. It devolves upon the doctor to teach.

It, therefore, interests us to know how milk becomes contaminated; so that we may better understand the prevention.

Ordinary bacteria (most of them) grow readily in milk. It is an excellent culture medium. In a test made; a petri-dish exposed under an udder during milking for 1 minute obtained nearly 5000 colonies of bacteria.

The method of contamination with the pathogenic organisms has already been extensively referred to:—Tuberculosis, Typhoid Fever, Scarlet Fever, Diphtheria, etc. This is fully understood and its prevention becomes easy.

The introduction of non-pathogenic bacteria into the milk comes from the stable air, hands and clothing of the milkers, dirt falling from the udder, or belly or tail of the cow into the pail, and from the excreta. These non-pathogenics when present in large numbers impair the nutritive value of milk and cause serious disease in weak, puny infants.

Bacterial standards are difficult to establish because of the wide diversity of opinion—some 10,000, others permit 30,000 bacteria per cubic centimeter.

Generally speaking, it is considered that 30,000 or under in bottled milk is good—and 10,000 or under excellent—while 100,000 for milk sold in cans is good. The Walker-Gordon Farm at Plainfield, N. J., a model of its kind—averaged for a year 5000, when delivered to customers 16 hours after milking. This is an exception. Good milk from other dairies has run from 10,000 to 100,000. From mixed dairies—the abomination of the milk question—milk delivered in cans has run as low as 100,000 and high as 40 million, in hot weather.

At the Walker-Gordon farm just referred to, the conditions under which milk is produced are as follows:—

Cement floors in stables readily cleansed—no dry food, hay, straw or fodder kept in stables, nor fed during milking—shavings for bedding—(not straw)—cows cleaned every day—udders kept thoroughly clean—milkers wear sterilized coats and caps and hands are washed prior to milking each cow—utensils sterilized with steam, milk removed at once, strained, mixed, cooled to 38, bottled and sealed 20 minutes from time it leaves cow, and is in New York and Philadelphia in two hours.

The conclusions at which we arrive, therefore, name several conditions as necessary in cow's milk in order to render it fit for food:

1. Fresh—not over 12 hours old before entirely consumed.

2. Clean—no dirt. Ordinary bacteria not above 100,000.

3. No pathogenic organisms.

4. Must be from healthy cows—no tuberculosis or other disease.

5. The food should be clean and fresh; no waste products.

6. Neither skimmed nor diluted.

7. Must contain no preservatives—as boric acid, salicylic acid or formaldehyde.

8. It should be at once placed and kept by the consumer in glass or porcelain vessels, tightly covered and in a refrigerator or other cold place, at a temperature of 50 degrees—better, 40 degrees.

These conditions are fulfilled in the observance of the set of rules governing the production and handling of milk set forth below, and generally accepted by the best authorities:

1. Clean cows. Clean the belly, udder and tail before milking, with water.

2. Milk out of doors, or in a place built for that purpose, preferably not in a stable.

"3. No fodder, hay or other dry food fed while milking. No dust raised by sweeping then or before.

"4. Milk must be handled away from dwellings, closets or stables. All washing of pails, cans, strainers, etc., at the dairy only and never removed from there. Milker's hands thoroughly cleansed just before milking.

"5. The sale of milk by people who have contagious disease at home strictly prohibited.

"6 No exposure to contagious disease, either through an individual thus suffering, or in contact with another. He should not enter a dairy nor have anything to do with milk or utensils.

"7. All dairies should be regularly inspected by Municipal or State Government. The tuberculin test regularly applied, and infected cattle from that or other disease, removed and use forbidden.

"8. Abolish dairy depots, and allow only the producer to sell milk, thus reducing the amount of handling, and holding some definite person responsible.

9. Ideally bottled at the dairy, first straining, cooling rapidly down to 45 deg. then bottled and kept at 45, delivered to the consumer as quickly as possible, pure, fresh and raw, neither pasteurized nor sterilized.

These rules do not require any great outlay of money, nor an expense disproportionate to the financial returns. Cleanliness costs little, and costly apparatus is not demanded.

Shall we have reform? Shall it be life or death for the children?

202-3 Peters Bldg.

---

DISCUSSION OF DR. CLARKE'S PAPER.

Dr. Claude Smith, City Bacteriologist, said the City Board of Health had taken active steps to regulate the supply of milk in order to have it fresh and pure.

They opposed cold storage and milk depots and thought they should be eliminated whenever possible. It is much better to have it direct from the dairymen. A report is to be made monthly to keep the public informed as to the quality of the milk sold by each dairy.

As regards tuberculosis in cattle, Dr. Smith thought it was not as frequent in the South as in the North, as they are out in the open air more of the time.

Milk should always be kept cold or the best of it will become contaminated and dangerous.

The board of health desires the co-operation of the physicians of Atlanta to help carry out the plans they have outlined, which will ensure for us the purest, freshest milk of any city of its size.

Dr. W. B. Armstrong emphasized the necessity for seeing that milk was properly cared for after leaving the dairyman and the physician should examine the refrigerators to see that there was plenty of ice.

Dr. K. L. Reid asked if it was necessary to deliver milk within a specified time in order to comply with the law.

Dr. Smith said no, that it was not practicable, but when it was shipped any distance it was required to be packed in ice.

Dr. Archibald Smith called attention to the carelessness with which certain milk depots had been operated.

Dr. Clarke (closing) said that if pasteurization of milk was to be effective it must be done immediately and that all handling increased the danger. Toxins in milk are not affected at all by pasteurization or sterilization. Spores are not destroyed.

Poor pasteurization is much worse than none at all, for the reason that it produces a false sense of security. All bacteria do not act upon milk-sugar, but upon the proteids, which may not produce sour milk, so as to warn the mother, and yet be poisonous.

Raw milk is the kind that should be sold. and this should be kept at 45 degrees or colder. A child should never be given milk that has been reheated when only a part has been taken previously.

Dr. W. A. Crowe said that in his opinion papers of this nature were public educators, and that the full force and effect of the paper would be lost unless it be given out to the world. Hetherefore moved that a committee be appointed to select such portions of the paper as were of value to the people of this city, and to publish the same in the daily press for the public benefit.

Dr. S. R. Roberts, in seconding this motion, said that one of the strongest statements made by Dr. Clarke, was the reference to the duties of the physician as a teacher, that much more of it should be done, and that he fully endorsed the movement to publish the paper.

